# Mini-Implants: New Possibilities in Interdisciplinary Treatment Approaches

**DOI:** 10.1155/2014/140760

**Published:** 2014-12-14

**Authors:** Biju Sebastian

**Affiliations:** Department of Orthodontics, Pushpagiri College of Dental Sciences, Thiruvalla, Kerala 689107, India

## Abstract

The introduction of mini-implants has broadened the range of tooth movements possible by fixed appliance therapy alone. The limits of fixed orthodontic treatment have become more a matter of facial appearance than anchorage. Many complex cases which would previously have required surgery or functional appliances can now be treated with fixed appliance therapy using mini-implants. A mutilated dentition case where mini-implants were used to provide anchorage for intrusion of molars and retraction of anterior teeth is reported here to illustrate this point.

## 1. Introduction

For years, orthodontic treatment has been limited in scope due to the range of tooth movements possible [1, 2]. Even within the limitations imposed as a result of this, anchorage was another issue which had to be tackled. The use of headgears, TPA, Nance palatal button to augment anchorage had its own set of problems. These severe restrictions led to the excessive use of functional appliances and orthognathic surgical procedures. But the introduction of mini-implants, miniplates, onplants, and so forth has brought about a paradigm shift in the field of orthodontics [3–9]. Many cases which need maximum anchorage for retraction or need intrusion/extrusion of anterior and posterior teeth can be treated now by fixed appliances when within reasonable limits with the help of mini-implants. This does not diminish the role played by functional appliances or orthognathic surgical procedures, but the introduction of mini-implants has revolutionized the field of orthodontics such that many more complex cases can now be treated with the help of fixed appliances alone. This paper is an attempt to illustrate this point with help of the following case.

## 2. Case Presentation

**Figure 1:**
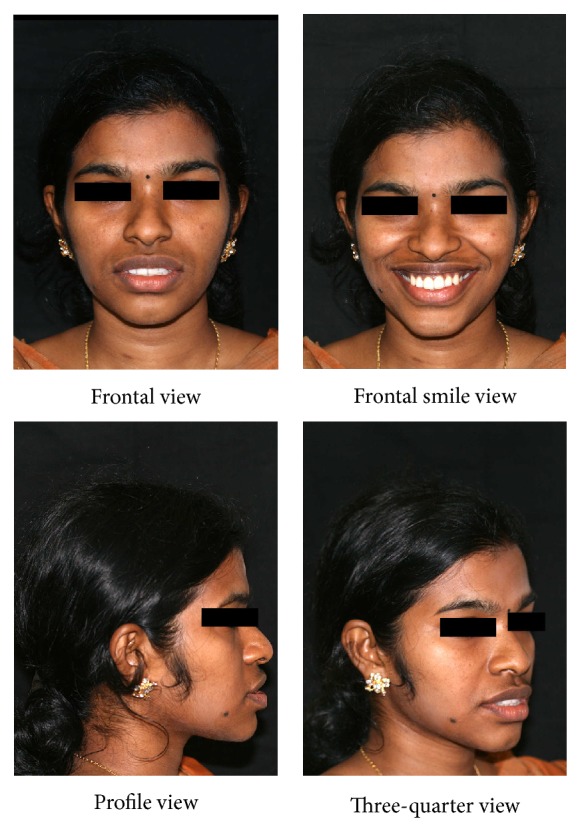
Pretreatment extraoral photographs.

**Figure 2:**
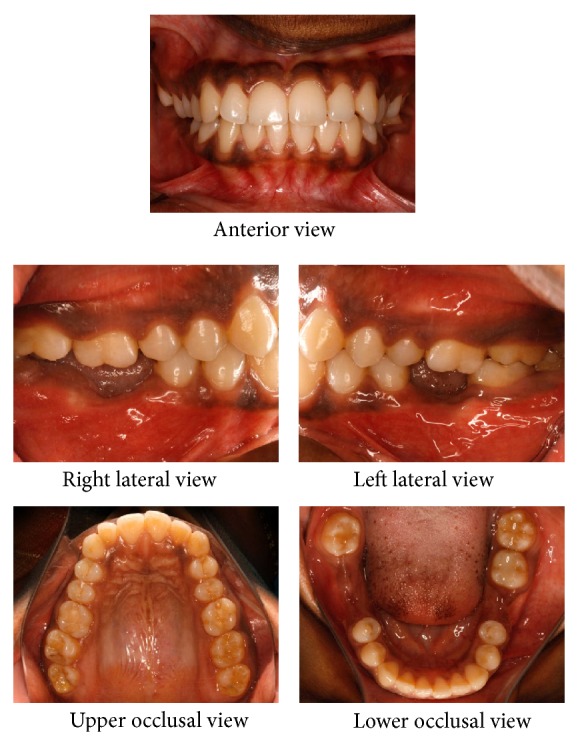
Pretreatment intraoral photographs.

A twenty-one-year-old female reported to the hospital for correction of protruding front teeth and replacement of missing lower back teeth. She had a Class I incisor relationship on a Class III skeletal base with an average maxillary-mandibular plane angle and increased lower face height (Figure 1). Intraoral examination revealed proclined upper anterior teeth and supraerupted upper first molars and upper right second molar due to missing lower first molars and lower right second molar (Figure 2). Bolton analysis showed a mandibular anterior tooth material excess of 1.8 mm.

**Figure 3:**
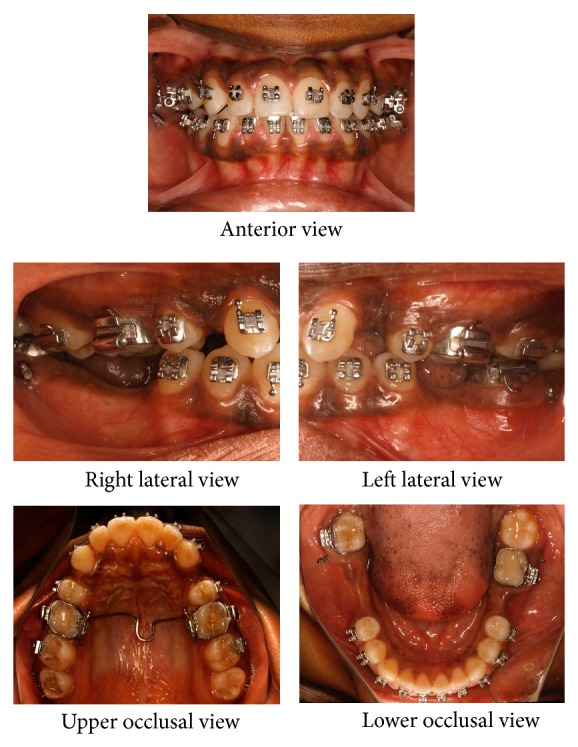
Mid-treatment intraoral photographs: alignment stage with microimplants for lower anterior retraction.

Treatment involved removal of upper first premolars and placement of mini-implants for lower anterior retraction and upper right second molar intrusion. The logic behind using mini-implants for lower anterior retraction was for anchorage due to missing lower first molars and lower right second molar. In addition, intrusion of 17 was required for placement of artificial substitute in the lower arch considering the level of extrusion of 17. Mini-implants of 1.3 mm × 8 mm (Absoanchor Mini-Implant, Dentos Inc., Daegu, Korea) were used for this purpose. The implants were positioned palatally between 16 and 17 and buccally between 17 and 18. The palatal implant was placed 7 mm superior to gingival margin with care taken to avoid Greater Palatine Artery. The palatal mucosa was more fibrous and thicker than that on the buccal aspect.  Figure 3 shows the positioning of mini-implants placed for lower anterior retraction. Retraction of upper and lower anteriors was done on 0.019′′ × 0.025′′ stainless steel wire with hooks. Retraction force was derived from 12 mm medium force NiTi close coil springs in lower arch and active tiebacks in upper arch.

**Figure 4:**
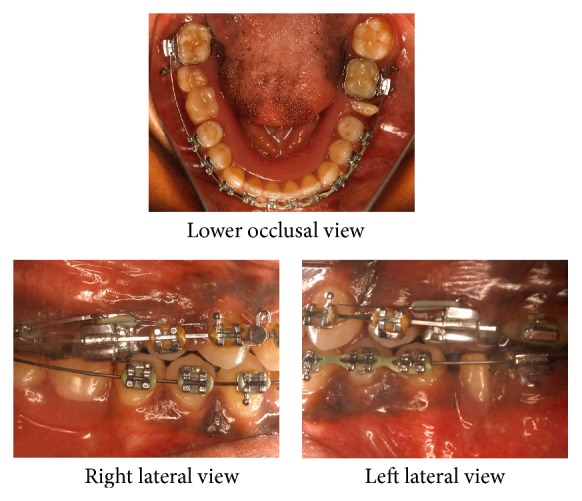
Mid-treatment intraoral photographs: with lower space maintainer.

**Figure 5:**
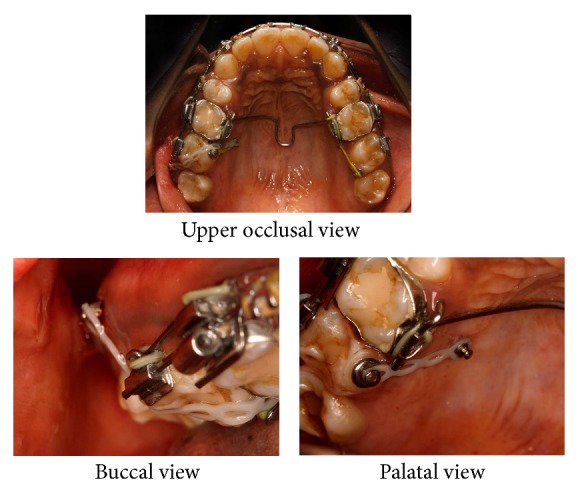
Mid-treatment intraoral photographs: with mini-implants for 17 intrusion. Intrusive force on 17 is provided by running an e-chain across the occlusal surface of 17. Further intrusive force on 17 from the lingual aspect is provided by an e-chain traction force to the lingual button on 17.

In the space closure stage, a lower removable partial denture was placed for space maintenance in the lower arch (Figure 4).  Figure 5 illustrates the mini-implants and mechanics used for 17 intrusion. Once intrusion of 17 was found to be adequate, active intrusive force on 17 was stopped and passive ligation was done to stabilize the correction achieved. The cephalometric assessment of the treatment result is illustrated in Table 1. During the course of the treatment, the upper anterior teeth were intentionally overangulated to mask the lower anterior tooth material excess of 1.8 mm. This led to unaesthetic appearance of upper incisors and dental midline deviation which was corrected by repositioning the brackets.

**Figure 6:**
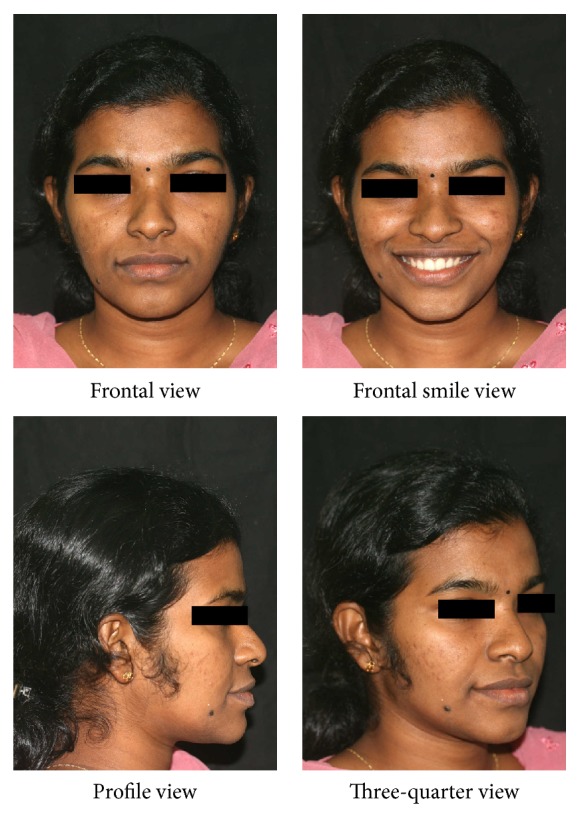
Posttreatment extraoral photographs.

**Figure 7:**
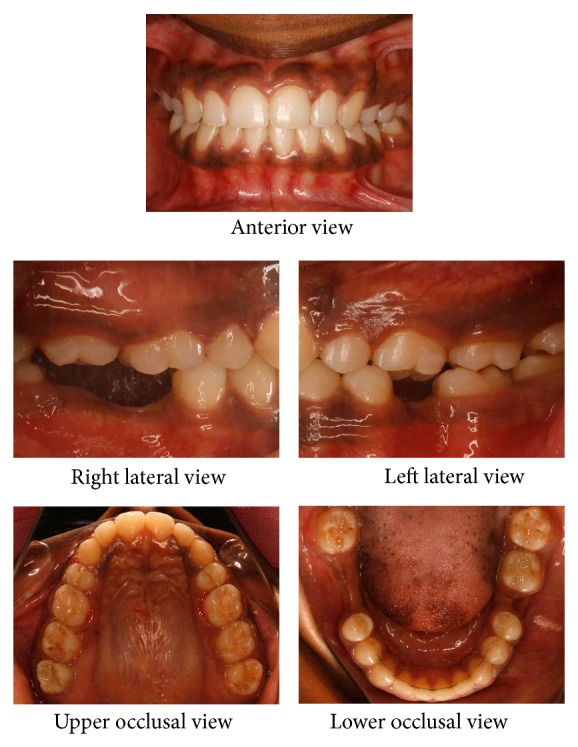
Posttreatment intraoral photographs: without removable cast partial denture.

**Figure 8:**
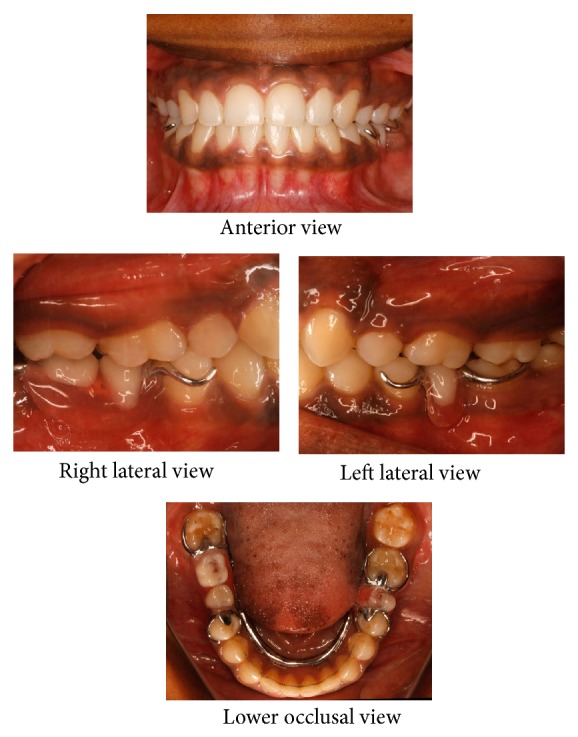
Posttreatment intraoral photographs: with removable cast partial denture.

Oral rehabilitation was completed with the placement of a lower cast partial denture replacing the missing lower molars (Figures 6, 7, and 8). Even though implant supported fixed crowns were suggested as a better option, the patient opted for a cast partial denture due to her time constraints and financial limitations.

## 3. Discussion

**Figure 9:**
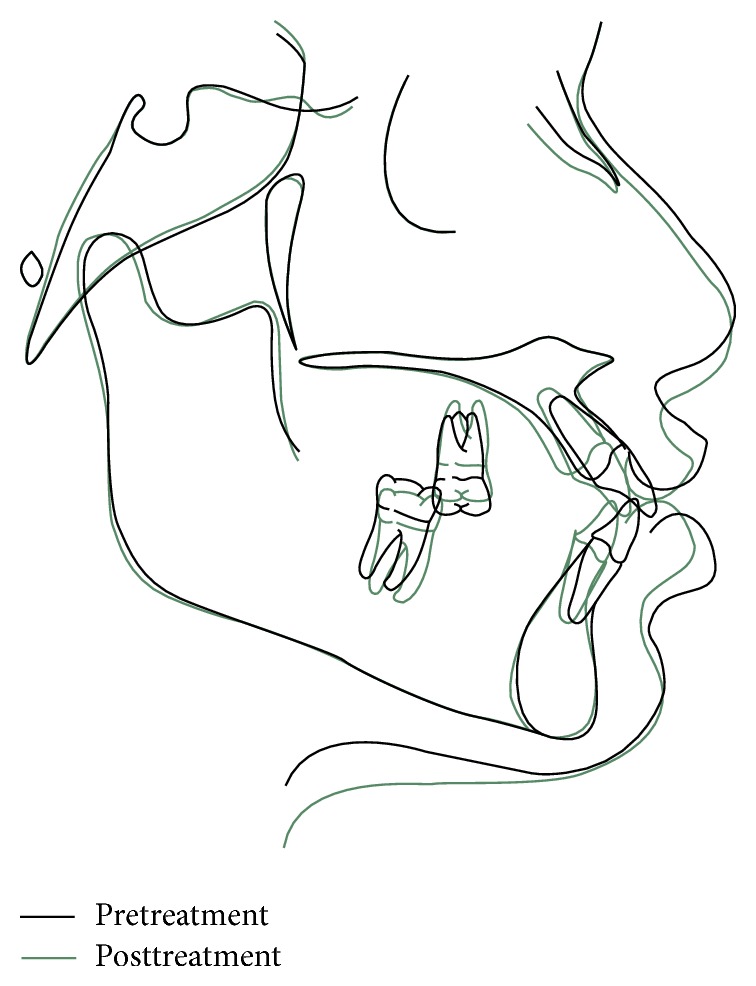
Cephalometric superimposition of pre- and posttreatment lateral cephalograms.

The advantages of using mini-implants in orthodontic treatment are clearly illustrated in the superimposed pre- and posttreatment cephalometric tracing of the above case report (Figure 9). Earlier treatment of such mutilated cases would have been near impossible with orthodontic therapy alone. On the lower right posterior segment, there is only third molar to provide anchorage for retraction which is not adequate in a normal circumstance. Also the level of extrusion of 17 would have meant that intentional RCT and crown would have to be done for completing the oral rehabilitation. Other methods of intrusion would not have been adequate considering the level of 17 extrusion. An added advantage of using mini-implants is that they are temporary anchorage devices that can be removed after treatment quite easily [10].

Quite a number of recent publications illustrate the use of mini-implants for other types of tooth movements like molar distalization [11] and protraction [12] and as anchor units for palatal expansion [13] or extraoral force application [14–16]. Since the introduction of mini-implants is a recent feature, we have not yet ascertained the extent to which all these are possible. But the guidelines for these will come with experience in the near future, eventually leading to a broadening of the envelope of discrepancy for tooth movement possible orthodontically [17].

## Figures and Tables

**Table 1:** Pre- and posttreatment cephalometric values.

Variable	Pretreatment	Posttreatment	Change
SNA	86°	86°	0°
SNB	86°	85°	−1°
ANB	0°	1°	1°
N perpendicular to A point	1 mm	1 mm	0 mm
N perpendicular to pogonion	2 mm	2 mm	0 mm
Go-Po	76 mm	76 mm	0 mm
SN to maxillary plane	6°	6°	0°
Wits appraisal	−2 mm	−1 mm	1 mm
Upper incisor to maxillary plane angle	130°	122°	−8°
Lower incisor to mandibular plane angle	95°	90°	−5°
Interincisal angle	111°	126°	15°
16 to maxillary plane	23 mm	21 mm	−2 mm
MM angle	24°	24°	0°
Upper anterior face height	44 mm	44 mm	0 mm
Lower anterior face height	58 mm	58 mm	0 mm
Face height ratio	57%	57%	0%
Lower incisor to APo line (linear value)	8 mm	4 mm	−4 mm
Lower lip to Ricketts E Plane	4 mm	0 mm	−4 mm
